# Carcinome urothélial invasif vésical récidivant traité par cystectomie avec enterocystoplastie iléale de substitution: à propos d'un cas

**DOI:** 10.11604/pamj.2019.33.184.17917

**Published:** 2019-07-11

**Authors:** Tresor Kibangula Kasanga, Daniel Ilunga Ntanga, Eric Mbuya Musapoudi, Nathalie Dinganga Kapessa, Dimitri Kanyanda Nafatalewa, Vincent De Paul Kaoma Cabala, Mbey Mukaz

**Affiliations:** 1 Département de Chirurgie, Unité d'Urologie, Faculté de Médecine, Clinique Universitaire de Lubumbashi, Université de Lubumbashi, Lubumbashi, Province du Haut-Katanga, République Démocratique du Congo

**Keywords:** Carcinome urothelial vesical, cystectomie totale, enterocystoplastie ileale, Bladder urothelial carcinoma, total cystectomy, ileal enterocystoplasty

## Abstract

Notre travail vise à rapporter un cas de carcinome urothelial vésical qui est une tumeur rare chez les sujets de moins de 40 ans, et apporter notre expérience thérapeutique, cela à travers l'observation d'un patient âgé de 37 ans, avec antécédent d'infection urinaire et un séjour dans une région riveraine (bilharziose) ayant consulté pour hypogastralgie et hématurie totale macroscopique. Le bilan clinique et paraclinique, mettait en évidence une volumineuse masse tumorale vésicale d'allure infiltrante, sans envahissement ganglionnaire ou organique local ou à distance. Sa prise en charge chirurgicale a consisté en une exérèse biopsique tumorale vésicale suivie d'une cystectomie totale, avec enterocystoplastie iléale de substitution. Les suites opératoires ont été simples. Il avait bénéficié également d'une chimiothérapie adjuvante et une vitaminothérapie B12. Les contrôles cliniques et paracliniques effectués 6 et 12 mois ne montraient aucune récidive.

## Abstract (en)

We here report a case of bladder urothelial carcinoma, a rare tumor in subjects less than 40 years, as well as our therapeutic experience. The study involved a 37-year old patient with a history of urinary tract infection and of staying at a riparian area (bilharzia) presenting with hypogastralgia and total macroscopic hematuria. Clinical and paraclinical assessment showed voluminous bladder mass suggesting infiltrative tumor without local or distant lymph node or organic involvement. Surgical management was based on resection of bladder tumor and biopsy followed by total cystectomy with substitutive ileal enterocystoplasty. The postoperative course was uneventful. The patient also underwent adjuvant chemotherapy and vitamin B-12 therapy. Clinical and paraclinical assessment, performed after 6 and 12 months, showed no recurrence.

## Introduction

Les tumeurs de la vessie surviennent généralement chez le sujet âgé entre 50 et 70 ans [1]. L'atteinte chez le jeune adulte (moins de 40 ans) est rare et ne dépasse pas 4% selon les séries [2]. L'évolution naturelle et le pronostic du carcinome à cellules transitionnelles (CCT) de la vessie chez les jeunes patients restent un sujet de débat et peu de données ont été publiées concernant cette entité. Nous présentons un cas de carcinome urothelial vésical chez un patient agé de 37 ans dans le but d'apporter notre expérience au sujet de sa prise en charge dont le résultat est encourageant.

## Patient et observation

**Figure 1:**
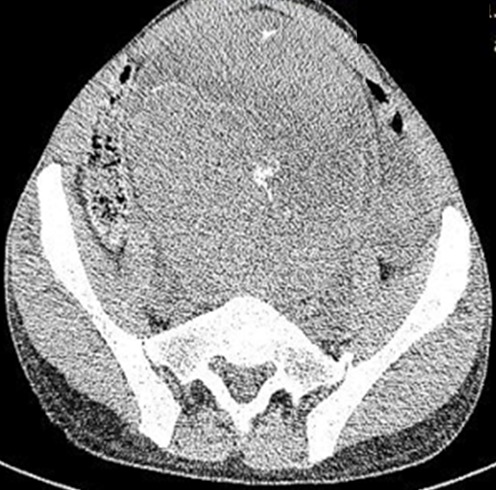
Image du scaner abdominal montrant la masse tumorale vesicale

Nous vous rapportons le cas d'un patient âgé de 37 ans admis au service d'urologie des Cliniques Universitaires de Lubumbashi, pour hypogastralgie, remontant à environ une année. La survenue d'une hématurie macroscopique totale en cours suivie de mictalgie depuis deux semaines avait motivé sa consultation. Dans ses antécédents; nous avons relevé la notion de séjour dans une région riveraine (bilharziose), notion d'infection urinaire itérative, pas de contage à la tuberculose, non tabagique, pas de notion des tumeurs des voies urinaires dans la famille. Au complément d'anamnèse; douleur et tuméfaction hypogastrique etaient d'apparition progressive accompagnée d'hématurie en cours; pollakiurie; mictalgie; pas de dysurie; pas de fièvre. A l'examen physique son état général était marqué par l'amaigrissement. L'examen de l'abdomen a révélé une voussure hypogastrique médiane à convexité tournée vers le haut; non sensible; de consistance ferme de surface irrégulière: à la pression de laquelle elle ne donne aucune envie d'uriner; à l'épreuve de 3 verres l'hématurie était totale a renforcement terminale; le toucher pelvien était sans particularité. Le diagnostic de tumeur vésicale a été retenu; confirmé par le scanner abdominal ce dernier avait mis en évidence une volumineuse masse tumorale pelvienne Figure 1, a priori intra vésicale, de 19 cm de grand axe, et s'accompagnant d'une urétéro-hydronéphrose bilatérale à prépondérance gauche, déjà relativement chronique. Le bilan d'extension semble par contre négatif aux niveaux ganglionnaire, hépatique et osseux. Le bilan hématologique réalisé avait montré, l'Hémoglobine= 6,4g%, Hématocrite= 21%, Globule rouge= 2 850 000/mm^3^, Plaquettes= 860 000/mm^3^, Globule Blanc= 12 680/mm^3^, Vitesse de Segmentation= 110 mm/h, Temps de Saignement= 2'00", Temps de coloration= 4'00", Groupe Sanguin = O Rh+, Formule Leucocytaire: Granulocyte= 69%, Lymphocyte= 20%, Monocyte= 11%, VGM= 74,2 mm^3^, TGM Hb = 22,6pg, CGM Hb= 30,5%.

**Figure 2:**
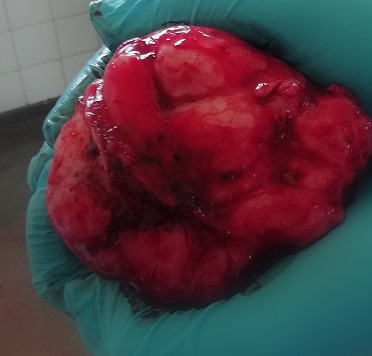
Macroscopie de la tumeur vessicale

L'échographie abdomino-pelvienne avait mis en évidence un foie homogène de volume normal, a contours réguliers, pas de masse aorte. Estomac, pancréas sans particularité. Rate homogène, volume normal. Les deux reins présentent des cavités anéchogènes communicantes à gauche, le bassinet et uretère sont dilatés. Vessie augmentée de volume, présente trois masses solides reliées entre elles par des cloisons ayant des contours irréguliers et ponctués. Les deux antérieures grosses masses portent du bas fond vésical. La postérieure mesure environ 140x87 mm et l'antérieure environ 97x43 mm. Elles présentent des flux vasculaires internes. Prostate difficile à visualiser. Conclusion: carcinomes vésicaux, probables compliqués d'hydronéphrose bilatérale (grade A à droite et B à gauche). Le scanner abdominal a mis en évidence une volumineuse masse tumorale pelvienne, a priori intra vésicale, de 19cm de grand axe, et s'accompagnant d'une urétéro-hydronéphrose bilatérale à prédominance gauche. Déjà relativement chronique. La radiographie du thorax et autres examens n'avaient rien de particulier. Dans un premier temps, nous avions réalisé une tumorectomie vésicale Figure 2 et urétérostomie cutanée bilatérale iliaquetemporaire et avons également pris la masse, une portion de la paroi vésicale antérieure et la portion juxta vésicale de l'uretère pour une analyse anatomo-pathologique. En période post-opératoire, il a été pris en charge par une équipe mixte associant les Réanimateurs et les Chirurgiens. Il avait bénéficié d'une antibiothérapie (ciprofloxacine flacon 500 mg: 2x500 mg pendant 7 jours après antibiogramme) et d'un apport liquidien en perfusion (Sérum Glucosé 10% 1 L pendant 8 heures, Sérum physiologique 1L pendant 8 heures, Sérum Ringer Lactate 1 L pendant 8 heures) et une analgésie (Perfac*: Paracétamol infusion 100 mg S/2x1 infusions en IVD pendant 30 minutes) par voie parentérale, une transfusion de deux unités de sang de poche de 450^°^C.

**Figure 3:**
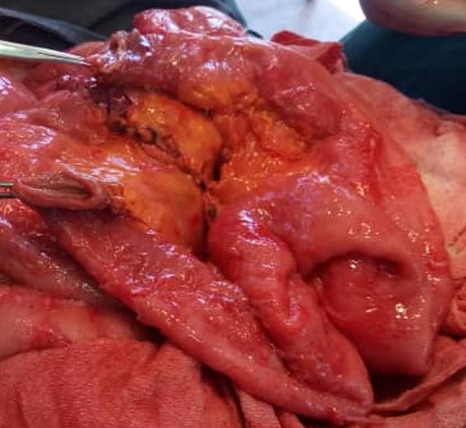
Segment iléal prélevé avant la confection de la néo poche vesicale

**Figure 4:**
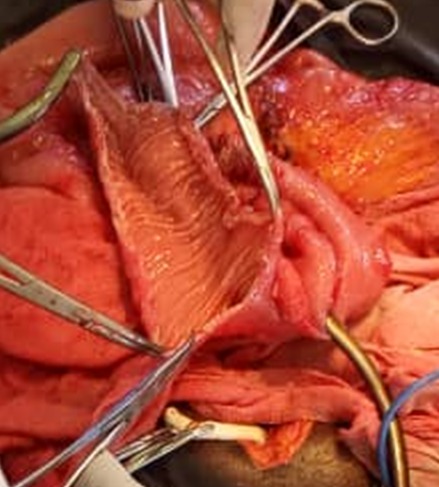
Confection de l'enterocystoplastie ileale de substitution

Les suites post-opératoires étaient marquées par une récidive de la masse vésicale deux semaines après, accompagnée d'hématurie totale, de douleur hypogastrique et d'insuffisance rénale au sixième jour post-opératoire. La biopsie avait mis en évidence un Carcinome urothelial vésicale invasif. Nous avions obtenu un consentement libre et éclairé après un cunsilling, nous avons préparé le tube digestif (Dulcolax comprimé 5mg peros, lavement évacuateur pendant 5 jours, régime pauvre en résidus. Un bilan préopératoire a été réalisé et nous avons repris le patient. Ce dernier a bénéficié d'une Cystectomie totale et enterocystoplastie iléale de substitution en W de Ghoneim Figure 3 et Figure 4 avec curage ganglionnaire sans evidence d'extension. Nous avons laissé une sonde nasogastrique et une sonde vésicale à 3 voies en place pendant 10 jours. Nous avons également réalisé le bilan sept jours après l'intervention, l'urée est redevenue à 14mg%, créatinine à 0,60mg%. Eu égard aux résultats, nous l'avions soumis sous nutrition parenterale, on procédait au rinçage de la vessie avec 50°C de sérum physiologique chaque jour pour liberer les mucus produits par la muqueuse iléale, au bout duquel l'ablation de la sonde se faite et le patient uriné seul sans problème. Après la cicatrisation de la plaie, son état général est redevenu bon, nous avons fait un bilan précure et nous l'avons soumis sous chimiothérapie adjuvante (Cisplatine et 5 Fluorouracile) après avis d'oncologue et une vitaminothérapie B12 parentérale 1000mg par mois.

## Discussion

La cystectomie radicale avec enterocystoplastie selon bricker représente le gold standard dans le traitement des tumeurs vésicales infiltrat depuis 1950 [3]. Le carcinome urothelial de la vessie survient essentiellement à partir de la cinquantaine [1]. Avant 40 ans, cette pathologie est rare et ne dépasse pas 4% selon les séries [1]. Selon Mc Carty, la fréquence du CCT de la vessie avant 30 ans est estimée à 0, 8% [4]. La prédominance masculine est nette dans toutes les séries [1]. Comme c'était le cas pour notre patient qui était du sexe masculin agé de 37 ans. Certains auteurs ne notent pas de différence significative de la survenue de carcinome urothelial chez le sujet jeune et le sujet adulte de plus de 40 ans [1]. L'étiologie de cette tumeur vésicale reste inconnue toute fois, pour certains auteurs, il existe des facteurs favorisants, une prédisposition héréditaire dans la survenue des tumeurs vésicales chez le sujet jeune, et des mutations génétiques survenant sur les chromosomes 7 et 17 sont incriminées, résultats qui sont encore préliminaires [5]. L'infection urinaire chronique entraine une irritation de la muqueuse vésicale responsable des lésions metaplasiques et dysplasiques conduisant a une tumeur urothelial vésicale [6]. Une hypothèse pathogénique plus récente concerne la catalyse de certaines bactéries Gram négatif avec transformationde nitrates en nitrites puis en nitrosamines potentiellementcarcinogènes [7]. Outre la bilharziose, à l'origine d'une irritation vésicale chronique liée aux infections récidivantes et à l'altération de la paroi par le parasite, ce qui rejoint notre observation, le patient avait des infections urinaires a répétitions et avait un séjour dans une région riveraine. Le tabac est également un facteur de risque reconnu.

Les neurovessies ont une incidence de cancers élevée [6], et certains autres facteurs favorisants ont été évoqués: le cyclophosphamide [8], avec une incidence proportionnelle à la durée du traitement et à la dose totale; le rôle des papillomavirus est discuté [9]. Ce qui n'a pas été observé chez notre patient. Par rapport à la récidive, pas de différence significative entre le sujet de moins ou de plus de 40ans dans la plupart des séries [4], ce qui fut le cas dans notre observation. Mais la majorité d'auteurs s'accordent à dire que la tumeur du sujet de moins de 30 ans est typiquement superficielle, monofocale, de petite taille et dotée d'un faible taux de récidive [6]. Le traitement du carcinome urothelial vésical est essentiellement chirurgical, mais encore non codifié. Il repose sur une cystectomie partielle, pour les petites tumeurs superficielles [6] et une cystoprostatectomie radicale chez l'homme ou une pelvectomie antérieure chez la femme en cas de tumeurs infiltrantes. Certains auteurs préconisent une urètrectomie au cours du geste [8] et une chimiothérapie adjuvante est basée sur les protocoles, ces derniers procèdent de façon empirique l'association cisplatine et 5 FU(5-fuorouracile) étant actuellement la plus utilisée essentiellement dans les tumeurs infiltrantes et en cas d'envahissement ganglionnaire, d'autres la réservent pour les formes métastatiques [8]. Chez notre patient nous avons plutôt réalisé une cystectomie totale et une enterocystoplastie iléale de substitution mais avec conservation de la prostate, cette dernière n'était pas infiltrée par rapport au bilan d'extension réalisé, et une réimplantation urétérale était réalisée. Keenan dans son étude en 2015 montre que 38% des patients ont développé en post enterocystoplastie iléale le déficit en vitamine B12, anémiemégaloblastique, trouble neuropsychiatrique, neuropathie périphérique par demyelinisation [10]. Notre patient avait été soumis à une vitaminothérapie B12 et aucune complication n'avait été mise en évidence.

## Conclusion

En dépit du caractère hautement malin du carcinome urothelial vésical une chirurgie adaptée sélective par entérocystoplastie de subtitution iléale pratiquée précocement, selon les cas de figure permettrait une qualité de vie satisfaisante après chirurgie et un recul au-delà de 12 mois par surveillance carcinologique.

## Conflits d’intérêts

Les auteurs ne déclarent aucun conflit d'intérêts.
